# The role of the epithelial Na^+^ channel (ENaC) in high AVP but low aldosterone states

**DOI:** 10.3389/fphys.2012.00304

**Published:** 2012-07-31

**Authors:** James D. Stockand

**Affiliations:** Department of Physiology, University of Texas Health Sciences Center, San AntonioTX, USA

**Keywords:** adrenal insufficiency, SIADH, sodium transport, hypertension, hyponatremia, diabetes insipidus, sodium excretion

## Abstract

Due to the abundance of seminal discoveries establishing a strong causal relation between changes in aldosterone signaling, the activity of the epithelial Na^+^ channel (ENaC) and blood pressure, the role of ENaC in health and disease is understood almost exclusively through the concept that this channel functions (in the distal nephron) as a key end-effector controlling renal sodium excretion during feedback regulation of blood pressure by the renin-angiotensin-aldosterone system (RAAS). Recent findings of aldosterone-independent stimulation of ENaC by vasopressin challenge the completeness of dogmatic understanding where ENaC serves solely as an end-effector of the RAAS important for control of sodium balance. Rather the consequences of activating ENaC in the distal nephron appear to depend on whether the channel is activated in the absence (by aldosterone) or presence [by vasopressin (AVP)] of simultaneous activation of aquaporin 2 water channels. Thus, a unifying paradigm has ENaC at the junction of two signaling systems that sometimes must compete: one controlling and responding to changes in sodium balance, perceived as mean arterial pressure, and the other water balance, perceived as plasma osmolality.

In many instances, particularly during hyponatremia and hypernatremia, plasma sodium likely contributes to the control of blood pressure. As is clear when considering the action of diuretics that suppress tubular sodium reabsorption, plasma sodium, and thus blood pressure, is influenced by renal sodium excretion. Renal sodium excretion is fine-tuned in the aldosterone-sensitive distal nephron (ASDN). The epithelial Na^+^ channel (ENaC) is expressed in the apical plasma membrane of principal cells of the ASDN (Garty and Palmer, 1997; Kellenberger and Schild, 2002). Here, ENaC serves as the primary cell entry pathway for electrogenic Na^+^ reabsorption from the urine back into interstitial fluid. Consequently, the activity of ENaC is limiting for trans-cellular sodium reabsorption across the ASDN.

Normal ENaC function is required for proper sodium balance and thus, normal blood pressure. Gain-of-function mutations in ENaC cause inappropriate renal sodium retention and consequent increases in mean arterial pressure (Lifton et al., 2001; Rossier et al., 2002). Inhibition of ENaC corrects the renal and blood pressure phenotypes resulting from such mutations. Loss-of-function mutations in ENaC, in contrast, cause renal sodium wasting and corresponding decreases in blood pressure (Rossier et al., 2002; Geller, 2005).

As a key end-effector modulating blood pressure, the activity of ENaC in the ASDN, as depicted in (the right of) Figure 1 is under negative-feedback regulation by the renin-angiotensin-aldosterone system (RAAS). The mineralocorticoid, aldosterone, is the final hormone in this cascade. This anti-natriuretic factor is essential for proper Na^+^ balance (Horisberger and Diezi, 1983). Decreases in blood pressure evoke via renin-AngII signaling secretion of aldosterone from the adrenal gland. Aldosterone through the mineralocorticoid receptor (MR) stimulates ENaC in the ASDN to minimize renal sodium excretion in protection of plasma sodium (Rossier et al., 2002; Geller, 2005). Pathological increases in aldosterone elevate blood pressure by promoting inappropriate renal sodium retention (White, 1994; Gomez-Sanchez, 1998). Inhibition of ENaC ameliorates this. In contrast, pathological decreases in aldosterone result in sodium wasting arising from inappropriate increases in renal sodium excretion (White, 1994; Geller, 2005). MR agonism and antagonism increase and decrease ENaC activity, respectively (Kemendy et al., 1992; Pácha et al., 1993; Palmer et al., 1994). Simply put, there is strong support for a tight positive relation between the levels and actions of aldosterone and ENaC activity, renal sodium reabsorption and blood pressure. In this sense, activation of ENaC is anti-natriuretic protecting plasma sodium and blood pressure.

**Figure 1:**
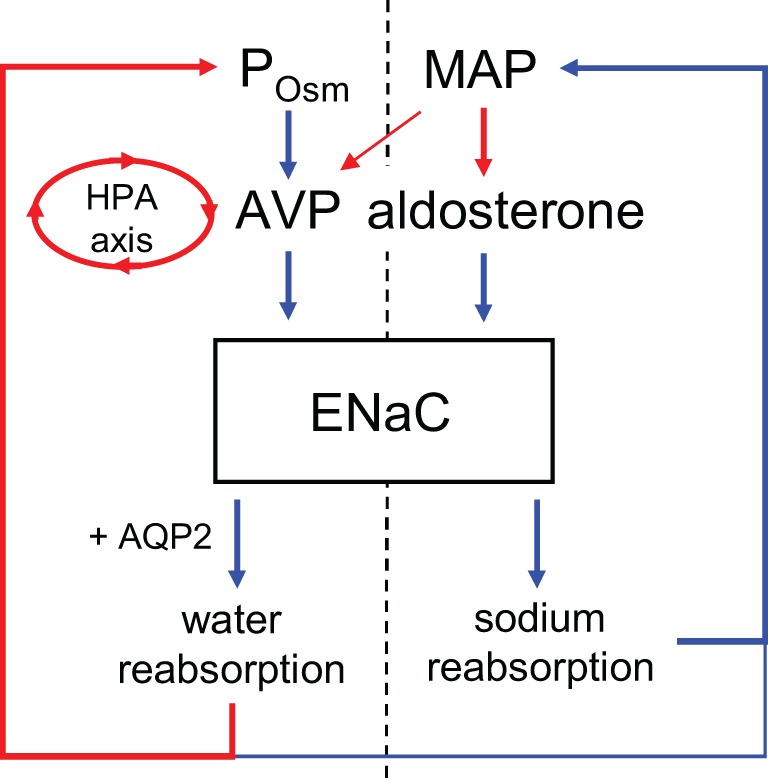
**Regulation of ENaC by aldosterone and vasopressin**. Vasopressin release is stimulated primarily by increases in plasma osmolality but also by decreases in mean arterial pressure and is also under feedback control by the hypothalamic-pituitary-adrenal (HPA) axis as mediated by glucocorticoids. Positive and negative relations are indicated by blue and red, respectively. Weaker regulation indicated with narrower arrows.

Because of the abundance of seminal discoveries establishing this strong causal relation between changes in aldosterone, ENaC activity and blood pressure, the role of ENaC in health and disease is understood almost exclusively through the concept of feedback regulation by the RAAS (Garty and Palmer, 1997; Lifton et al., 2001; Rossier et al., 2002; Geller, 2005). Emerging evidence, though, argues that this dogma is inadequate too narrowly defining the role of ENaC in sodium and water balance. For instance, ENaC activity is high, in some instances, in the absence of significant changes in aldosterone (Palmer et al., 1994; Mironova et al., 2012). This form of regulation is termed aldosterone-independent in the current review. Several hormones in addition to aldosterone modulate the activity of ENaC. Vasopressin (AVP) decreases renal sodium excretion by increasing the activity of ENaC and sodium reabsorption in the ASDN in parallel with aldosterone (Bankir, 2001; Perucca et al., 2008; Stockand, 2010; Blanchard et al., 2011). Indeed, both AVP and aldosterone are required to achieve maximal urinary concentration: minimization of free water excretion.

The antidiuretic hormone, AVP, increases free water reabsorption by stimulating aquaporin 2 water channels in the distal nephron (Bankir, 2001; Fenton and Knepper, 2007). The axial corticomedullary hyperosmotic gradient as established by the loop of Henle provides the draw for this AQP2-mediated water reabsorption. In addition to being anti-aquaretic, AVP is anti-natriuretic (Helman et al., 1971; Frindt and Burg, 1972; Andersen et al., 1990; Bankir et al., 2005). As extrapolated from rich but indirect evidence, ENaC, as shown in (the left of) Figure 1, appears to be a target for AVP in this regard. AVP stimulates sodium reabsorption in isolated perfused ASDN in an amiloride-sensitive manner, and amiloride inhibits the anti-natriuretic actions of AVP in man (Helman et al., 1971; Frindt and Burg, 1972; Reif et al., 1984; Tomita et al., 1986; Schlatter and Schafer, 1987; Hawk et al., 1996). Amiloride is a blocker of ENaC. Moreover, V_2_ receptor antagonism promotes sodium excretion in addition to water excretion (Andersen et al., 1990; Bankir, 2001; Bankir et al., 2005; Perucca et al., 2008). The V_2_ receptor mediates AVP actions in the ASDN (see Stockand, 2010 for a recent review of AVP signaling and actions on ENaC in the ASDN). AVP also increases the trans-epithelial voltage difference across isolated ASDN consistent with it activating electrogenic transport processes (Helman et al., 1971; Frindt and Burg, 1972; Reif et al., 1984; Schlatter and Schafer, 1987; Hawk et al., 1996). Cell entry of sodium across the apical membrane via ENaC, as discussed above, is the limiting step in electrogenic sodium reabsorption in the ASDN. Moreover, AVP increases the activity of ENaC in cultured immortalized renal cells (Marunaka and Eaton, 1991). Recently, we demonstrated that AVP increases the activity of ENaC in freshly isolated, split-open murine ASDN (Bugaj et al., 2009).

As discussed above, regulation of plasma sodium and thus, volume is considered to be under the purview of the RAAS with ENaC playing a critical role in this process; and plasma osmolality under the control of feedback regulation by AVP with AQP2 being the critical end-effector in this process. However, the compelling support for AVP-activation of ENaC suggests that these paradigms need to be broadened to incorporate the concept that activation of ENaC in the presence of activated AQP2 contributes to the draw for water reabsorption out of the ASDN. This would be consistent with the function of ENaC (in the presence of activated AQP2) being shifted by AVP from protecting plasma sodium to facilitating water reabsorption. This exciting idea raises several questions centered on the hypothesis that the function of ENaC in the ASDN (with respect to systemic sodium and water balance) dependents on whether the channel is activated in the absence or presence of simultaneous activation of AQP2 water channels where AVP activates both ENaC and AQP2.

Upon initial consideration, the idea that AVP-activated ENaC facilitates free water reabsorption seems counter-intuitive to the established role for this channel in protecting plasma sodium. However, the essential question here is whether ENaC activated in the ASDN in the presence of AQP2 contributes more to the draw for free water reabsorption or plasma [Na^+^]: a question that largely remains to be answered. Insight, though, can be gleamed from several recent findings as discussed below.

Addressing whether ENaC activated in the presence of active AQP2 by AVP influences the draw for free water more than plasma [Na^+^] is significant for it will define the paradigm (refer to Table 1) by which we understand the role of ENaC in hyponatremic and hypernatremic states of low aldosterone but high AVP. Elaboration of this question, moreover, is significant because it also will define the limitations of having two distinct feedback systems, one responsive to and controlling plasma [Na^+^]/volume and the other plasma osmolality/water content, competing using a common final effector, ENaC (refer to Table 1).

**Table 1:** **The consequences of active ENaC in states of high AVP and low aldosterone**.

**State**	**AVP**	**Aldo**	**ENaC activity**	**Volume**	**P_Na_**	**Potential role of ENaC decreasing excretion of^a^**	
						**Sodium**	**Free water**
Adx	↑	↓	Robust	↓	↓	Compensatory to volume	Causal to hyponatremia
SIADH	↑	↓	?	–	↓	Compensatory to hyponatremia	Causal to hyponatremia

a The role of ENaC will depend on whether it has a relatively greater effect on decreasing renal sodium excretion vs. free water excretion.

## Aldosterone signaling through MR is sufficient but is it necessary for ENaC activity?

All studies investigating the actions of aldosterone on (amiloride-sensitive) renal sodium excretion, transport and the activity of ENaC in the ASDN are in agreement that increases in aldosterone are sufficient to increase ENaC activity (Pácha et al., 1993; Palmer et al., 1994). Decreased amiloride-sensitive sodium excretion resulting from gain-of-function mutations in MR and increases in aldosterone signaling also are in agreement on this point (White, 1994; Gomez-Sanchez, 1998; Geller, 2005).

The strong inverse relation between sodium intake and aldosterone levels and ENaC activity are consistent with aldosterone being necessary for the activity of this channel. In agreement are findings that MR antagonism decrease ENaC activity and increase renal sodium excretion (Pácha et al., 1993; Palmer et al., 1994). Moreover, recent results form genetically modified mice lacking MR or aldosterone synthase (AS) also support this position. MR null mice do not survive long after the first week of life without sodium supplementation due to pathological renal sodium excretion (Berger et al., 1998). Similarly, AS null mice have pronounced renal sodium and water wasting (Lee et al., 2005; Makhanova et al., 2006). Sodium restriction exacerbates renal salt and water wasting in both AS and MR null mice as compared to control animals, which have appropriate feedback regulation of ENaC by RAAS. These observations are reminiscent of human infants carrying inactivating MR mutations and AS deficiency, which require sodium supplementation to survive (White, 1994, 2004; Geller, 2005).

However, this issue is more complex than the above findings would seem to suggest. It was demonstrated recently that the inverse relation between sodium intake and ENaC activity is not solely dependent on aldosterone signaling (Toney et al., 2012), and neonatal MR null mice retain residual but significant ENaC activity, approximately 24% of normal, as extrapolated from amiloride-sensitive fractional Na^+^ excretion and transport across isolated, perfused collecting ducts (Berger et al., 1998). Such results are not consistent with aldosterone being an absolute requirement for ENaC activity in the ASDN. The strongest support for this, though, comes from recent findings that ENaC is expressed and active in the absence of aldosterone in adrenalectomized mice (Mironova et al., 2012). Thus, there are some instances where ENaC is active in the absence of aldosterone signaling. These exceptions then demonstrate that aldosterone is not necessary for the activity of ENaC in the ASDN. This raises the questions of what is maintaining ENaC activity high in the absence of aldosterone in these circumstances and what are the consequences of aldosterone-independent activation of ENaC.

## Aldosterone-independent activation of ENaC by AVP in hyponatremic states

The first clue to answering these questions comes from understanding that the hormonal state and thus phenotype of loss-of-function of MR or AS does not completely overlap that of adrenalectomy. With MR dysfunction, the RAAS is up-regulated (Berger et al., 1998). With AS dysfunction, aldosterone is absent but the levels of other adrenal steroids capable of mineralocorticoid action, in particular corticosterone, are increased (White, 2004; Lee et al., 2005). With adrenalectomy, there is no aldosterone or other adrenal steroids and catecholamines and thus, these animals receive no input from the adrenal gland to ENaC. Moreover, akin to adrenal insufficiency, removal of the adrenal glands causes marked increases in plasma AVP levels (Friedman et al., 1962b; Ahmed et al., 1967; Ishikawa and Schrier, 2003). This is not present with either MR or AS dysfunction (Makhanova et al., 2006).

Usually, AVP release is primarily controlled by plasma osmolality. Elevated AVP release in adrenal insufficient states (that lack both glucocorticoids and mineralocorticoids) results form two events. There is loss of negative-feedback regulation by glucocorticoids of the hypothalamic-pituitary axis controlling AVP release and there is strong non-osmotic stimulation of AVP release resulting from volume depletion due to sodium and water wasting by the kidney (Ahmed et al., 1967; Ishikawa and Schrier, 2003).

The second clue comes from recognizing that adrenal insufficiency and central diabetes insipidus are counterpoints when considering the equilibrium distribution of sodium and water: the pattern of sodium and water distribution in either deficiency depends in part on the activity of the remaining gland. As such, the hyponatremia of adrenal insufficiency is absent when combined with neurohypophyseal deficiency and in the Brattleboro rat, which has central diabetes insipidus (Friedman et al., 1962a,b; Foy and Schnieden, 1965; Ahmed et al., 1967). Thus, the hyponatremia of adrenal insufficiency is dependent on elevated AVP release.

As discussed above, AVP is known to decrease renal Na^+^ excretion by increasing reabsorption in the ASDN by stimulating ENaC activity. Akin to its regulation of AQP2 water channels, AVP stimulates ENaC in principal cells via the V_2_ receptor (Bugaj et al., 2009; Stockand, 2010). Inhibition of the V_2_ receptor decreases ENaC activity in the ASDN of adrenaletomized mice to levels that are identical to those observed in control animals (Mironova et al., 2012). This demonstrates that elevated AVP is the driving force maintaining ENaC activity high in the absence of aldosterone in adrenalectomized mice. Could this aldosterone-independent AVP-stimulation of ENaC (in the presence of activated AQP2) be contributing to the draw of water out of the collecting duct and thus, be contributing to the hyponatremia common to adrenal insufficiency? As depicted in Table 1 only additional research will clarify this issue.

To appreciate the potential consequences of aldosterone-independent activation of ENaC by AVP, it must be recognized that with adrenalectomy ENaC is no longer regulated in a normal manner by feedback signaling in response to changes in sodium balance. This is a result of stimulation by elevated AVP release combined with disruption of regulation by the RAAS. Rather in adrenal insufficient states, ENaC is regulated primarily by AVP: a hormone that normal controls plasma osmolality.

Adrenalectomy, similar to other adrenal insufficient states, though, causes pronounced renal sodium and volume wasting; likely through loss of a non-ENaC, aldosterone-dependent but AVP-insensitive mechanism (Mironova et al., 2012). This muddies the waters with respect to whether AVP-dependent stimulation of ENaC in adrenal insufficiency is a compensatory response to decreases in volume or a result independent of actions on vascular volume. It is noteworthy that AS and MR null mice also have significant renal salt and water and thus, volume loss but the activity of ENaC under these conditions, which are not noted for significant increases in [AVP], is markedly less than that in adrenalectomized mice, which do have elevated [AVP]. Regardless of whether increases in ENaC activity are compensatory or not, a consequence of adrenal insufficiency is that AVP-stimulated ENaC, in the absence of input from RAAS, becomes a slave to water reabsorption rather than a key mediator of sodium balance possibly exacerbating the hyponatremia of this state. As outlined in Table 1, a definitive test of the role of AVP-activated ENaC during adrenal insufficiency will be to determine whether blockade of this channel betters or worsens the hyponatremia of this state. As yet these experiments have not been performed.

Unlike adrenal insufficiency, hyponatremia in the syndrome of inappropriate ADH (AVP) secretion (SIADH) is not associated with hypovolemia. No results are available currently regarding ENaC activity in this condition and thus, it is unclear if increases in AVP in this setting also stimulate ENaC. Prolonged agonism of the V_2_ receptor in a rat model of SIADH, though, increased ENaC abundance even in the absence of input from the adrenal gland (Ecelbarger et al., 2001; Tiwari et al., 2006). Unfortunately, no information exists currently regarding whether inhibition of ENaC betters or worsens hyponatremia in SIADH. Such studies are key for they will elaborate the effects of AVP-dependent stimulation of ENaC in hyponatremic states with and without confounding hypovolemia.

## Concluding remarks

While several key questions remain about the physiological and pathological consequences of AVP-stimulation of ENaC, it is clear that AVP can increase the activity of this channel. This positions ENaC to be an end-effector of both aldosterone, as the final signal in the RAAS and AVP. Signaling through RAAS, in part because it activates ENaC, influences plasma sodium and thus, blood pressure; and signaling by AVP influences free water reabsorption, in part, because it activates AQP2. As argued above, AVP-stimulated ENaC likely facilitates water reabsorption. This contributes to protection of vascular volume as likely is the case in adrenal insufficiency, but does it do so at the expense of facilitating hyponatremia? Such a question highlights the potential limitations imposed by the mechanics of water movement only through osmosis in biological systems and by the evolution of the mammalian renal tubule where ENaC sits at the intersection of two homeostatic control systems one responding to and influencing plasma sodium, and the other plasma osmolality that must by their nature sometimes be in competition.

### Conflict of interest statement

The author declares that the research was conducted in the absence of any commercial or financial relationships that could be construed as a potential conflict of interest.

## References

[B1] Ahmed A. B. George B. C. Gonzalez-Auvert C. Dingman J. F. (1967). Increased plasma arginine vasopressin in clinical adrenocortical insufficeincy and its inhibition by glucosteroids. J. Clin. Invest. 46, 111–123 10.1172/JCI105504 6018744 PMC297026

[B2] Andersen L. J. Andersen J. L. Schutten H. J. Warberg J. Bie P. (1990). Antidiuretic effect of subnormal levels of arginine vasopressin in normal humans. Am. J. Physiol. 259, R53–R60 2375429 10.1152/ajpregu.1990.259.1.R53

[B3] Bankir L. (2001). Antidiuretic action of vasopressin: quantitative aspects and interaction between V1a and V2 receptor-mediated effects. Cardiovasc. Res. 51, 372–390 10.1016/S0008-6363(01)00328-5 11476728

[B4] Bankir L. Fernandes S. Bardoux P. Bouby N. Bichet D. G. (2005). Vasopressin-V2 receptor stimulation reduces sodium excretion in healthy humans. J. Am. Soc. Nephrol. 16, 1920–1928 10.1681/ASN.2004121079 15888562

[B5] Berger S. Bleich M. Schmid W. Cole T. J. Peters J. Watanabe H. Kriz W. Warth R. Greger R. Schutz G. (1998). Mineralocorticoid receptor knockout mice: pathophysiology of Na^+^ metabolism. Proc. Natl. Acad. Sci. U.S.A. 95, 9424–9429 9689096 10.1073/pnas.95.16.9424 PMC21354

[B6] Blanchard A. Frank M. Wuerzner G. Peyrard S. Bankir L. Jeunemaitre X. Azizi M. (2011). Antinatriuretic effect of vasopressin in humans is amiloride sensitive, thus ENaC dependent. Clin. J. Am. Soc. Nephrol. 6, 753–759 10.2215/CJN.06540810 21233458 PMC3069366

[B7] Bugaj V. Pochynyuk O. Stockand J. D. (2009). Activation of the epithelial Na^+^ channel in the collecting duct by vasopressin contributes to water reabsorption. Am. J. Physiol. 297, F1411–F1418 10.1152/ajprenal.00371.2009 19692483 PMC2781343

[B8] Ecelbarger C. A. Knepper M. A. Verbalis J. G. (2001). Increased abundance of distal sodium transporters in rat kidney during vasopressin escape. J. Am. Soc. Nephrol. 12, 207–217 11158210 10.1681/ASN.V122207

[B9] Fenton R. A. Knepper M. A. (2007). Mouse models and the urinary concentrating mechanism in the new millennium. Physiol. Rev. 87, 1083–1112 10.1152/physrev.00053.2006 17928581

[B10] Foy J. M. Schnieden H. (1965). The effects of endocrine gland ablation and of corticosteroid substitution on the rate of water turnover in the adrenalectomized rat. J. Endocrinol. 31, 89–94 10.1677/joe.0.0310089 14241739

[B11] Friedman S. M. Sreter F. A. Nakashima M. Friedman C. L. (1962a). Adrenal cortex and neurohypophyseal deficiency in salt and water homeostasis of rats. Am. J. Physiol. 203, 697–701 13959820 10.1152/ajplegacy.1962.203.4.697

[B12] Friedman S. M. Sreter F. A. Nakashima M. Friedman C. L. (1962b). Pitressin or aldosterone effects in rats with adrenal and neurohypophyseal deficiency. Am. J. Physiol. 203, 702–708 13959821 10.1152/ajplegacy.1962.203.4.702

[B13] Frindt G. Burg M. B. (1972). Effect of Vasopressin on Sodium transport in renal cortical collecting tubules. Kidney Int. 1, 224–231 4671214 10.1038/ki.1972.32

[B14] Garty H. Palmer L. G. (1997). Epithelial sodium channels: function, structure, and regulation. Physiol. Rev. 77, 359–396 9114818 10.1152/physrev.1997.77.2.359

[B15] Geller D. S. (2005). Mineralocorticoid resistance. Clin. Endocrinol. (Oxf.) 62, 513–520 10.1111/j.1365-2265.2005.02229.x 15853818

[B16] Gomez-Sanchez C. E. (1998). Primary aldosteronism and its variants. Cardiovasc. Res. 37, 8–13 10.1016/S0008-6363(97)00230-7 9539852

[B17] Hawk C. T. Li L. Schafer J. A. (1996). AVP and aldosterone at physiological concentrations have synergistic effects on Na^+^ transport in rat CCD. Kidney Int. Suppl. 57, S35–S41 8941920

[B18] Helman S. I. Grantham J. J. Burg M. B. (1971). Effect of Vasopressin on electrical resistance of renal cortical collecting tubules. Am. J. Physiol. 220, 1825–1832 5087832 10.1152/ajplegacy.1971.220.6.1825

[B19] Horisberger J.-D. Diezi J. (1983). Effects of mineralocorticoids on Na^+^ and K^+^ excretion in the adrenalectomized rat. Am. J. Physiol. 245, 89–99 6869541 10.1152/ajprenal.1983.245.1.F89

[B20] Ishikawa S. E. Schrier R. W. (2003). Pathophysiological roles of arginine vasopressin and aquaporin-2 in impaired water excretion. Clin. Endocrinol. (Oxf.) 58, 1–17 10.1046/j.1365-2265.2003.01647.x 12519405

[B21] Kellenberger S. Schild L. (2002). Epithelial sodium channel/degenerin family of ion channels: a variety of functions for a shared structure. Physiol. Rev. 82, 735–767 10.1152/physrev.00007.2002 12087134

[B22] Kemendy A. E. Kleyman T. R. Eaton D. C. (1992). Aldosterone alters the open probability of amiloride-blockable sodium channels in A6 epithelia. Am. J. Physiol. 263(Pt 1), C825–C837 1329547 10.1152/ajpcell.1992.263.4.C825

[B23] Lee G. Makhanova N. Caron K. Lopez M. L. Gomez R. A. Smithies O. Kim H. S. (2005). Homeostatic responses in the adrenal cortex to the absence of aldosterone in mice. Endocrinology 146, 2650–2656 10.1210/en.2004-1102 15731365

[B24] Lifton R. P. Gharavi A. G. Geller D. S. (2001). Molecular mechanisms of human hypertension. Cell 104, 545–556 10.1016/S0092-8674(01)00241-0 11239411

[B25] Makhanova N. Sequeira-Lopez M. L. Gomez R. A. Kim H. S. Smithies O. (2006). Disturbed homeostasis in sodium-restricted mice heterozygous and homozygous for aldosterone synthase gene disruption. Hypertension 48, 1151–1159 10.1161/01.HYP.0000249902.09036.e7 17075030

[B26] Marunaka Y. Eaton D. C. (1991). Effects of vasopressin and cAMP on single amiloride-blockable Na channels. Am. J. Physiol. Cell Physiol. 260, C1071–C1084 1852105 10.1152/ajpcell.1991.260.5.C1071

[B27] Mironova E. Bugaj V. Roos K. P. Kohan D. E. Stockand J. D. (2012). Aldosterone-independent regulation of the epithelial Na channel by vasopressin in adrenalectomized mice. Proc. Natl. Acad. Sci. U.S.A. 109, 10095–10100 10.1073/pnas.1201978109 22665796 PMC3382497

[B28] Palmer L. G. Antonian L. Frindt G. (1994). Regulation of apical K and Na channels and Na/K pumps in rat cortical collecting tubule by dietary K. J. Gen. Physiol. 104, 693–710 7836937 10.1085/jgp.104.4.693 PMC2229228

[B29] Pácha J. Frindt G. Antonian L. Silver R. B. Palmer L. G. (1993). Regulation of Na channels of the rat cortical collecting tubule by aldosterone. J. Gen. Physiol. 102, 25–42 8397276 10.1085/jgp.102.1.25 PMC2229165

[B30] Perucca J. Bichet D. G. Bardoux P. Bouby N. Bankir L. (2008). Sodium excretion in response to vasopressin and selective vasopressin receptor antagonists. J. Am. Soc. Nephrol. 19, 1721–1731 10.1681/ASN.2008010021 18596120 PMC2518442

[B31] Reif M. C. Troutman S. L. Schafer J. A. (1984). Sustained response to vasopressin in isolated rat cortical collecting tubule. Kidney Int. 26, 725–732 6097738 10.1038/ki.1984.208

[B32] Rossier B. C. Pradervand S. Schild L. Hummler E. (2002). Epithelial sodium channel and the control of sodium balance: interaction between genetic and environmental factors. Annu. Rev. Physiol. 64, 877–897 10.1146/annurev.physiol.64.082101.143243 11826291

[B33] Schlatter E. Schafer J. A. (1987). Electrophysiological studies in principal cells of rat cortical collecting tubules. ADH increases the apical membrane Na^+^-conductance. Pflugers Arch. 409, 81–92 2441357 10.1007/BF00584753

[B34] Stockand J. D. (2010). Vasopressin regulation of renal sodium excretion. Kidney Int. 78, 849–856 10.1038/ki.2010.276 20736986

[B35] Tiwari S. Packer R. K. Hu X. Sugimura Y. Verbalis J. G. Ecelbarger C. A. (2006). Increased renal alpha-ENaC and NCC abundance and elevated blood pressure are independent of hyperaldosteronism in vasopressin escape. Am. J. Physiol. Renal Physiol. 291, F49–F57 10.1152/ajprenal.00390.2005 16449357

[B36] Tomita K. Pisano J. J. Burg M. B. Knepper M. A. (1986). Effects of Vasopressin and Bradydkinin on anion transport by the rat cortical collecting duct. J. Clin. Invest. 77, 136–141 10.1172/JCI112268 3080471 PMC423319

[B37] Toney G. M. Vallon V. Stockand J. D. (2012). Intrinsic control of sodium excretion in the distal nephron by inhibitory purinergic regulation of the epithelial Na(+) channel. Curr. Opin. Nephrol. Hypertens. 21, 52–60 10.1097/MNH.0b013e32834db4a0 22143248 PMC3689579

[B38] White P. C. (1994). Disorders of aldosterone biosynthesis and action. N. Engl. J. Med. 331, 250–258 10.1056/NEJM199407283310408 8015573

[B39] White P. C. (2004). Aldosterone synthase deficiency and related disorders. Mol. Cell. Endocrinol. 217, 81–87 10.1016/j.mce.2003.10.013 15134805

